# Astrocytes derived from trisomic human embryonic stem cells express markers of astrocytic cancer cells and premalignant stem-like progenitors

**DOI:** 10.1186/1755-8794-3-12

**Published:** 2010-04-27

**Authors:** Sailesh Gopalakrishna-Pillai, Linda E Iverson

**Affiliations:** 1 Stem Cell Biology, Beckman Research Institute of the City of Hope, 1450 E. Duarte Road, Duarte CA, 91010, USA

## Abstract

**Background:**

Trisomic variants of human embryonic stem cells (hESCs) arise spontaneously in culture. Although trisomic hESCs share many properties with diploid hESCs, they also exhibit features of cancer stem cells. Since most hESC-based therapies will utilize differentiated derivatives, it is imperative to investigate the potential of trisomic hESCs to undergo malignant transformation during differentiation prior to their use in the clinical setting.

**Methods:**

Diploid and trisomic hESCs were differentiated into astrocytic progenitors cells (APCs), RNA extracted and hybridized to human exon-specific microarrays. Global gene expression profiles of diploid and trisomic APCs were compared to that of an astrocytoma cell line and glioblastoma samples, analyzed by others, using the same microarray platform.

**Results:**

Bioinformatic analysis of microarray data indicates that differentiated trisomic APCs exhibit global expression profiles with similarities to the malignant astrocytoma cell line. An analogous trend is observed in comparison to glioblastoma samples indicating that trisomic APCs express markers of astrocytic cancer cells. The analysis also allowed identification of transcripts predicted to be differentially expressed in brain tumor stem cells. These data indicate that *in vitro *differentiation of trisomic hESCs along astrocytic pathways give rise to cells exhibiting properties of premalignant astrocytic stem/progenitor cells.

**Conclusions:**

Given their occult nature, opportunities to study premalignant stem/progenitor cells in human have been few. The ability to propagate and direct the differentiation of aneuploid hESCs provides a powerful *in vitro *system for investigating biological properties of human cells exhibiting features of premalignant stem cells. This *in vitro *culture system can be used to elucidate changes in gene expression occurring enroute to malignant transformation and to identify molecular markers of cancer stem/progenitor cells. These markers are invaluable for diagnostic purposes and may be novel targets for therapeutic intervention.

## Background

Human embryonic stem cells (hESCs) are a source of pluripotent cells that can be differentiated *in vitro *into cells of numerous lineages [1]. The use of hESCs in regenerative medicine requires caution since aneuploid variants of hESCs spontaneously arise in culture. Trisomy for chromosomes X, 12 and/or 17 is one type of aneuploidy frequently observed in hESC lines [2]. Trisomic hESC variants exhibit many properties indistinguishable from their diploid counterparts; they self-renew, express 'stem' markers characteristic of diploid hESCs, retain pluripotency, differentiate *in vitro *and produce teratomas in mice [3,4]. Trisomic variants appear karyotypically stable over time in culture and microarray and RT-PCR analyses indicate that gene expression patterns of trisomic hESCs are similar to the diploid hESC lines from which they were derived [3,4]. However, trisomic hESC variants also display characteristics similar to cancer stem cells; they exhibit a reduced doubling time and teratomas arising from trisomic hESC injection contain a higher percentage of undifferentiated cells similar to teratocarcinomas formed following embryonal carcinoma cell injection [3]. Many similarities in gene expression profiles have been reported for normal and cancer stem cells, suggesting that changes in expression of relatively few genes may be sufficient to drive transformation of normal stem cells into cancer stem cells [5]. Recent evidence indicates that neural precursors derived from variant hESC lines exhibit early features of neoplastic transformation, including increased proliferative capacity and ~20 fold increase in the frequency of tumor initiating cells when assayed by injection into NOD-SCID mice [6]. Since cultured hESCs are genetically unstable and exhibit a propensity to develop spontaneous trisomy [2,7], it is imperative to evaluate the potential tumorigenicity of trisomic hESC variants.

In general, hESC-based cell-replacement strategies will rely on hESC-derived differentiated cells rather than the pluripotent stem cells. Thus, the potential of aneuploid hESC variants to undergo malignant transformation in the clinical setting is more instructively evaluated by comparing expression profiles of differentiated derivatives of diploid and trisomic hESCs. The primary objective of this study was to determine if similarities in gene expression patterns of diploid and trisomic pluripotent hESCs are retained following *in vitro *directed differentiation. To investigate this question, an *in vitro *culture system was developed in which hESCs were differentiated into homogenous populations of human astrocytic progenitor cells (APCs) suitable for global gene expression profiling using high-density exon-specific microarrays. If expression patterns of trisomic hESCs diverge from diploid hESCs following differentiation, then the next objective was to determine if trisomic derivatives exhibit expression profiles similar to malignant cell lines and/or primary tumor samples of the same lineage. Given the difficulty of isolating sufficient quantities of human premalignant progenitors for sophisticated molecular characterization, the final objective of this study was to determine if expression patterns of differentiated derivatives of aneuploid hESCs express markers of previously identified astrocytic cancer stem/progenitor cells. The results of this analysis indicate that *in vitro *differentiated astrocytes derived from a trisomic hESC line exhibit global gene expression profiles similar to astrocytomas and astrocytic cancer stem/progenitor cells. The results demonstrate that the combination of *in vitro *directed differentiation of hESCs, global gene expression profiling and robust bioinformatic analyses provides a powerful model system that can be used to identify differentially expressed biomarkers in stem/progenitor cells in heterogeneous tumors.

## Methods

### HESC and other cell culture

HESC lines H9 (WiCell) and BG01V (American Type Culture Collection) were grown under feeder-independent conditions on matrigel-coated dishes (BD) in medium containing basal DMEM/F-12 with 1 mM glutamine, 20% knockout serum replacement (KSR; Invitrogen), 2 mM non-essential amino acids and 8 ng/ml FGF (Invitrogen). To obtain non-adherent embryoid bodies, small pieces of undifferentiated hESC colonies (~100-200 cells) were mechanically dissected and cultured on low attachment plates in the same media used for maintaining pluripotent hESCs, except KSR was removed and replaced with 10% Fetal Bovine Serum (FBS; Hyclone). Neurospheres were derived from 4-5 day old embryoid bodies and grown in suspension for two weeks in medium containing DMEM/F-12 with 2 mM L-glutamine, 10 μl/ml BIT9500 (StemCell Technologies) supplemented with 10 ng/ml FGF, 10 ng/ml EGF (Millipore). To obtain astrocytic progenitor cells, neurospheres were allowed to adhere on matrigel-coated plates and differentiated in the presence of CCF-STTG1 conditioned media supplemented with 10 ng/ml EGF. CCF-STTG1 cells, a grade IV human astrocytoma cell line, were obtained from American Type Culture Collection and cultured in growth medium containing DMEM/F-12 with 2 mM L-glutamine, 1 mM sodium pyruvate, 4.5 g/l glucose, 1.5 g/l sodium bicarbonate supplemented with 10% FBS, under 5% CO_2 _at 37°C.

### Immunocytochemical characterization

Human ESCs grown on matrigel-coated LabTek chamber slides were rinsed with 1× PBS and fixed in 4% paraformaldehyde for 30 minutes at room temperature. Immunofluorescence staining was performed according to instructions provided in the Stem Cell Characterization Sample Kit (Millipore). The following primary antibodies were used at 1:200 dilutions: anti-TRA-1-60 (Millipore) and OCT-4 (Cell Signaling). Secondary antibodies, rhodamine-conjugated anti-mouse IgG or IgM or Cy2-conjugated anti-mouse IgM or IgG (Jackson Immunoresearch Laboratories Inc.) were used to detect primary antibody at 1:500 dilutions. H9 and BG01V APCs were cultured in matrigel-coated 24-well plates, the cells rinsed with 1× PBS and fixed in 4% paraformaldehyde for 30 minutes at room temperature. An astrocyte-specific primary antibody directed against glial fibrillary acidic protein (α-GFAP, Chemicon) was used at 1:200 dilution. Secondary antibody, rhodamine-conjugated anti-mouse IgG (Jackson Immunoresearch laboratories Inc.) was used to detect GFAP positive cells. Images were visualized with an Inverted IX81 (Olympus) fluorescence microscope and captured on a Retiga 2000R cooled CCD color camera (QImaging).

### Exon microarrays and data analysis

Affymetrix Human Exon 1.0ST microarrays were selected as the platform for global gene expression profiling because they contain 1.4 million probesets, including four oligonucleotides for each known or predicted exon in the human genome, and are expected to be more comprehensive than Affymetrix U133 Plus 2 microarrays, where most probesets are clustered in and around 3' regions of genes. Additional information can be obtained from the Affymetrix website http://www.affymetrix.com. RNA was isolated from three independent cultures of each cell type using the Trizol method (Invitrogen) and RNA quality determined by Nanodrop technique (NanoDrop Technologies). One μg of total RNA was used for preparation of individual samples. Microarray hybridization, scanning and data acquisition were performed at the City of Hope Microarray Core Facility. All microarray data were analyzed using Partek Genomic Suite software. Signal estimates obtained from CEL files were quantile-sketch normalized using the RMA algorithm for core probeset intensities. They were adjusted for "Detection Above Background", using surrogate GC mismatch intensities. Imported log2-transformed exon intensities were subjected to one-way ANOVA (analysis of variance) for comparison between the three biological replicates of each cell population, except glioblastoma samples where the 23 individual patient samples were treated as one cell population. Unsupervised hierarchical cluster analysis, heat maps and individual gene dot plots were generated using Partek Genomic Suite software. High-end (over) or low-end (under) expression is indicated by red and blue, respectively, in the heat maps. Expression level of each gene in each cell population was measured relative to the median expression level across the three replicates and variations between cell populations graphically depicted using individual gene dot plots, where the horizontal line within the colored bar represents the median expression level within each population and the vertical length of the bar represents SEM. Supervised hierarchical analyses, filtered at *p *value < 0.02 as described in detail in Results, was used to generate gene lists. Please see the Partek website http://www.partek.com/ for a more detailed description of statistical analysis of microarray data using the Partek Genomic Suite applications. Exon array data of 23 glioblastoma samples were extracted from GEO database: GSE9385 [8]. Exon microarray data of H9 APC, BG01V APC and CCF-STTG1 cells have been deposited at ArrayExpress http://www.ebi.ac.uk/arrayexpress: accession number is E-MEXP-2633.

### Semi-quantitative RT-PCR, DNA sequencing and quantitative RT-PCR analyses

H9 and BG01V hESCs cultured on matrigel were harvested after five to six days in culture. APCs were harvested on the fifth passage after neurospheres were differentiated into astrocytic progenitors on matrigel-coated plates. Total RNA was extracted using TRIzol according to the manufacturer's instructions (Invitrogen). Gene-specific PCR primers located within exons or spanning exon junctions were designed based on human gene sequences obtained from Ensembl http://www.ensembl.org/. Sequences of PCR primers are shown in Additional file 1, Table S1. Synthesis of cDNA was performed using 2 μg of total RNA, SuperScript II reverse transcriptase (Invitrogen) and random primers. Semi-quantitative RT-PCR reactions used 1 μl of cDNA template and exon-specific primers. PCR products were resolved by electrophoresis on 1.5% agarose gels and visualized by ethidium bromide staining. Data were recorded using QuantityOne software (BioRad). PCR product of interest was excised, purified and cloned into StrataClone vector using the PCR Cloning Kit (Stratagene) and the DNA insert sequenced using T3 or T7 primers. Quantitative PCR reactions (25 μl) were performed in an iCycler (BioRad) using 1 μl cDNA template, exon primers for the gene in question and SYBR green PCR mix (Applied Biosystems). Relative quantification was determined using the ΔΔCT method [9] according to the manufacturer's protocol (BioRad). Quantitative RT-PCR analysis using Human Cancer Pathway Finder PCR Arrays (SuperArray, Biosciences Corporation) containing proprietary RT-PCR primers for a number of cancer-associated genes was carried out according to the manufacturer's instructions using 1 μl cDNA template.

## Results

### Derivation of astrocytic progenitor cells from hESCs

**Figure 1:**
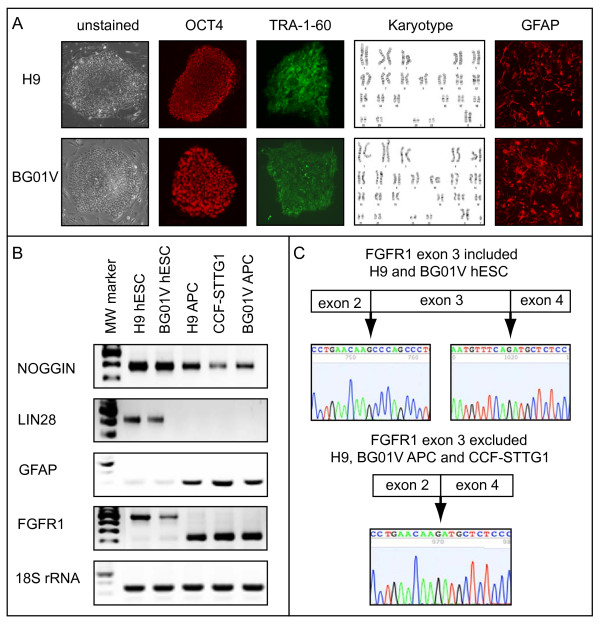
**Diploid and trisomic astrocytic progenitors derived by directed *in vitro *differentiation of hESCs exhibit transcript and alternative splicing patterns characteristic of astrocytic cells**. A. Diploid H9 and trisomic BG01V hESCs colonies (unstained, left panels), express stem cell markers OCT-4 (red) and TRA-1-60 (green). Karyotypes of diploid H9 (46; XX) and trisomic BG01V (49; XXY, +12, +17) hESCs. Diploid and trisomic APCs express astrocytic marker, GFAP (red). B. Semiquantitative RT-PCR analysis demonstrating that stem cell transcripts *Noggin *(top panel) and *Lin28 *(second panel) are over expressed in both diploid H9 and trisomic BG01V hESC lines relative to all astrocytic-like cells including H9 APCs, BG01V APCs and CCF-STTG1 astrocytoma cells. *GFAP *transcripts are up regulated in astrocytic cells relative to hESCs (middle panel). Alternative splicing pattern of *FGFR1 *in diploid H9 hESCs is preserved in trisomic BG01V hESCs relative to all astrocytic cells. 18S RNA was used as control in all RT-PCR experiments (bottom panel). MW marker is included in the far left lane in each gel. C. DNA sequence analysis demonstrating that the *FGFR1 *transcript expressed in diploid H9 and trisomic BG01V hESCs includes exon 3, while astrocytic cells express a *FGFR1 *splice variant in which exon 3 has been excluded (from *FGFR1 *RT-PCR analysis in Figure 1B).

Diploid H9 and trisomic BG01V hESCs were grown on mouse embryonic fibroblasts, transferred to matrigel and cultured under feeder-independent conditions for subsequent immunofluorescent and molecular characterization (Figure 1A). Both H9 and BG01V hESCs express OCT-4 and TRA-1-60 (Figure 1A) and other markers characteristic of pluripotent hESCs, including TRA-1-81 and SSEA-4 (not shown). Examples of H9 diploid karyotype (46; XX) and aneuploid BG01V karyotype (49; XXY, +12, +17) are shown in Figure 1A. H9 and BG01V hESCs were cultured under non-adherent conditions that promote differentiation into embryoid bodies and subsequent neurosphere formation as described in Methods. Floating hESC-derived neurospheres were allowed to settle on matrigel-coated dishes and differentiated into adherent astrocytic progenitor cells (APCs). Astrocytic nature of APCs was confirmed by staining for GFAP (Figure 1A) and additional molecular analyses described below. Semi-quantitative RT-PCR analysis demonstrates over expression of stem cell transcripts, *NOGGIN *and *LIN28*, in pluripotent H9 and BG01V hESCs relative to astrocytic-like cells (H9 APCs, BG01V APCs and CCF-STTG1 astrocytoma cells), while over expression of *GFAP *RNA is observed in all astrocytic-like cells relative to both hESC lines (Figure 1B). H9 and BG01V hESCs both express a *FGFR1 *mRNA including exon 3 (upper band), while all three astrocytic-like cells express a *FGFR1 *alternatively spliced variant in which exon 3 has been excluded from the mature transcript (lower band) (Figure 1B). Inclusion of *FGFR1 *exon 3 in pluripotent hESCs and exon 3 exclusion from astrocytic-like cells was confirmed by sequencing of the PCR product extracted from the gel (Figure 1C).

### Gene expression profile of trisomic APCs is similar to CCF-STTG1 astrocytoma cells

**Figure 2:**
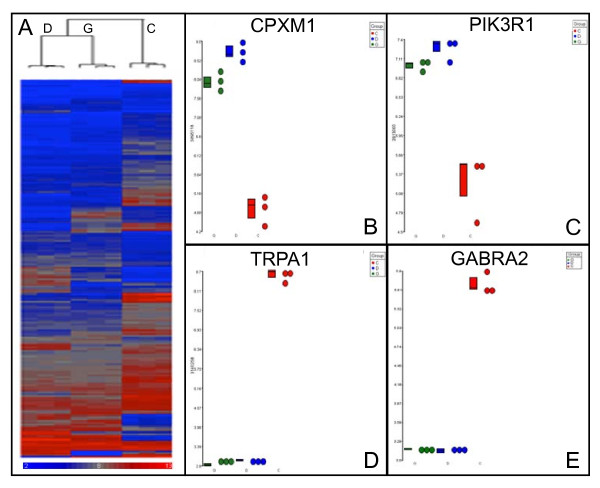
**Global gene expression profile of trisomic BG01V APCs is similar to CCF-STTG1 astrocytoma cells**. A. Heat map of unsupervised hierarchical cluster analysis of exon microarray hybridization data derived from the three diploid H9 APC samples (C), the three trisomic BG01V APC samples (G) and the three CCF-STTG1 astrocytoma cell samples (D). Relative over expression is indicated by red and under expression by blue in the heat map. Microarray data was filtered using a *p *value < 0.001. Individual gene dot plots showing changes in mean expression levels of transcripts *CPXM1 *(panel B), *PIK3R1 *(panel C), *TRPA1 *(panel D) and *GABRA2 *(panel E). Each panel shows the individual hybridization data for all nine samples, including the three biological replicates of diploid H9 APCs (red), the three independent replicates of trisomic BG01V APCs (green) and the three CCF-STTG1 astrocytoma cell samples (blue).

RNA samples extracted from three independent cultures of each of the three astrocytic-like cell populations (diploid H9 APCs, trisomic BG01V APCs and CCF-STTG1 astrocytoma cells) were used for microarray analysis. The nine RNA samples were subjected to linear amplification, labeling and hybridized to Affymetrix Human Exon 1.0ST microarrays. Statistical analysis of variance (ANOVA) of microarray data was performed using Partek Genomics Suite software applications as described in the Methods section. Excellent overall agreement among the three biological replicates of each cell type is readily apparent in the heat map generated by hierarchical clustering (Figure 2A), which displays all statistically significant (*p *value < 0.001) differences in relative expression levels between diploid H9 APCs (samples C), trisomic BG01V APCs (samples G) and CCF-STTG1 astrocytoma cells (samples D). This unsupervised hierarchical clustering indicates that the global gene expression profile of trisomic BG01V APCs shares many similarities with the malignant astrocytoma cells. Examples of log2-transformed, fold-changes in geometric mean expression levels of four transcripts are shown in Figure 2; two exhibiting significant over expression in trisomic BG01V APCs and CCF-STTG1 cells relative to H9 APCs, *CPXM1 *(Figure 2B) and *PIK3R1 *(Figure 2C), and two exhibiting significant under expression in trisomic BG01V APCs and CCF-STTG1 cells relative to H9 APCs, *TRPA1 *(Figure 2D) and *GABRA2 *(Figure 2E).

Numerous transcripts exhibit differences in relative expression levels (either over or under expression) in each of the three individual pair wise comparisons (GvC: 2929 differentially expressed transcripts, DvC: 4019 and GvD: 5193). Because the BG01V trisomic hESC line was not derived from the diploid H9 hESC line, and neither hESC line is related to the CCF-STTG1 astrocytoma cell line, many of these differences in expression levels undoubtedly arise from inherent genetic differences between the three distinct cell lines. It is for this reason that one cannot rely solely on the individual pair wise comparisons to identify the most statistically significant differences in gene expression. Rather, we focused on those gene transcripts exhibiting consistent sign changes in expression levels in both the BG01V APCs (G) and CCF-STTG1 astrocytoma cell line (D) relative to the H9 APCs (C) (i.e. both over expressed in BG01V APCs and CCF-STTG1 cells relative to H9 APCs or both under expressed in BG01V APCs and CCF-STTG1 cells relative to H9 APCs). Those transcripts exhibiting consistent sign changes in relative expression levels are identified by performing an analysis of variance (ANOVA) of the supervised hierarchical clustering; i.e. an ANOVA of the intersection of the two individual data sets (or the group comparison).

**Figure 3:**
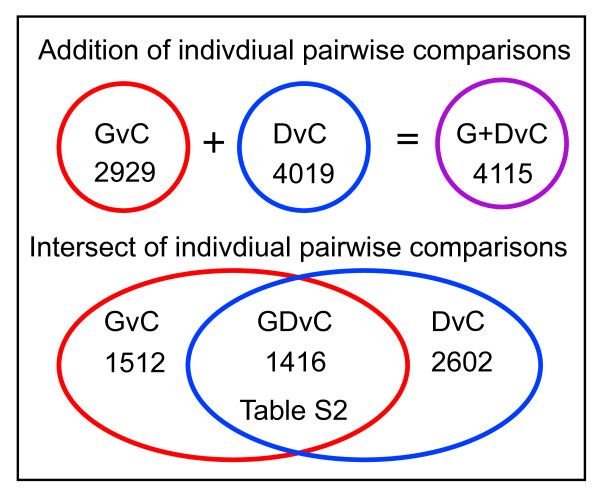
**Intersection of microarray data identifies common markers of astrocytic cancer cells**. Venn diagram depicting the overlap, identified by the supervised hierarchical analysis, between the two pair wise comparisons, GvC and DvC, that was used to identify the 1038 transcripts exhibiting consistent sign changes in expression patterns in trisomic BG01V APCs (samples G) and the CCF-STTG1 astrocytoma cell line (samples D) relative to diploid H9 APCs (samples C), which constitutes the GDvC gene list shown in Additional file 2, Table S2.

The power of the group comparison for refining the list of statistically significant differentially expressed transcripts is illustrated in Figure 3. The number of differentially expressed transcripts detected by ANOVA of the individual pair wise comparisons is 2929 for GvC and 4019 for DvC comparisons, and the total number of unique transcripts exhibiting differential expression in either the GvC or the DvC pair wise comparison - or the G+DvC data set - is 4115 (Figure 3, upper panel). However, the majority of these 4115 transcripts are not consistently over expressed (or consistently under expressed) in both BG01V APCs and CCF-STTG1 cells relative to H9 APCs. As indicated in Figure 3 (lower panel), transcripts exhibiting consistent sign changes in expression levels are identified by the group comparison (the intersect of the two data sets or the GDvC comparison). Once subjected to these more stringent criteria, the number of differentially expressed transcripts is reduced to only 1416. Thus, the group comparison is used to reduce the number of false positives that are scored as statistically significant in the individual pair wise comparisons (and which may arise solely from cell line specific differences in gene expression). These 'false positive' transcripts are eliminated because they do not meet the threshold of statistical significance as determined by ANOVA of the group comparison.

The complete list of 1416 transcripts that meet this higher threshold of significance is shown in Additional file 2, Table S2 (the GDvC comparison), where gene transcripts are listed in descending order on the basis of the combined average fold-change in expression levels in BG01V APC (samples G) and CCF-STTG1 astrocytoma cells (samples D) relative to H9 APCs (samples C). Although differences in mean expression levels of several transcripts are observed between BG01V APCs and CCF-STTG1 astrocytoma cells, visual inspection of the Additional file 2, Table S2 data set indicates that the ANOVA of the group comparison successfully eliminated most false positives. More than 96% of the 1,416 identified transcripts exhibit consistent sign changes; ~33% are over expressed and ~63% are under expressed in both BG01V APCs and CCF-STTG1 astrocytoma cells relative to diploid H9 APCs. Fewer than 4% of the transcripts show inconsistent sign changes (i.e. under expressed in BG01V APCs and over expressed in CCF-STTG1 cells relative to H9 APCs or over expressed in BG01V APCs and under expressed in CCF-STTG1 cells relative to H9 APCs). Most of the inconsistent transcripts exhibit only minor changes in relative expression levels (~10% to 20% increase or decrease) and relatively insignificant *p *values. In contrast, approximately 130 of the consistently over expressed transcripts show at least a two-fold increase in mean expression level in each individual pair wise comparison, BG01V APCs (samples G) vs. H9 APCs (samples C) and CCF-STTG1 (samples D) vs. H9 APCs (samples C), and far greater significance with *p *values less than ~2 × 10^-9^.

Many of the consistently and abundantly over expressed transcripts in trisomic BG01V APCs and CCF-STTG1 astrocytoma cells encode proteins previously implicated in cancer in general or associated with astrocytomas specifically, including *HSPA1A*, *HOXD10*, *GPNMB*, *GUCY1B3*, *GUCY1A3*, *HDAC9*, *APOE*, *CTSH*, *THRB*, *RAB38 *and *PIK3R1 *[10-21]. Transcripts exhibiting significant under expression in trisomic BG01V APCs and CCF-STTG1 astrocytoma cells relative to diploid H9 APCs include several markers of normal differentiated astrocytes, including *TRPA1*, *GABRA2, BDNF*, *BDKRB1 *and *BDKRB2 *[22-24]. This result suggests that directed differentiation of trisomic hESCs along an astrocytic lineage produces astrocytic progenitor cells with an intermediate phenotype; although BG01V APCs continue to express many biomarkers of normal, differentiated astrocytes (similar to diploid H9 APCs), they also express numerous markers that are characteristic of the malignant astrocytoma cell line (CCF-STTG1).

### RT-PCR validation of differentially expressed transcripts in trisomic and diploid hESC-derived APCs

**Figure 4:**
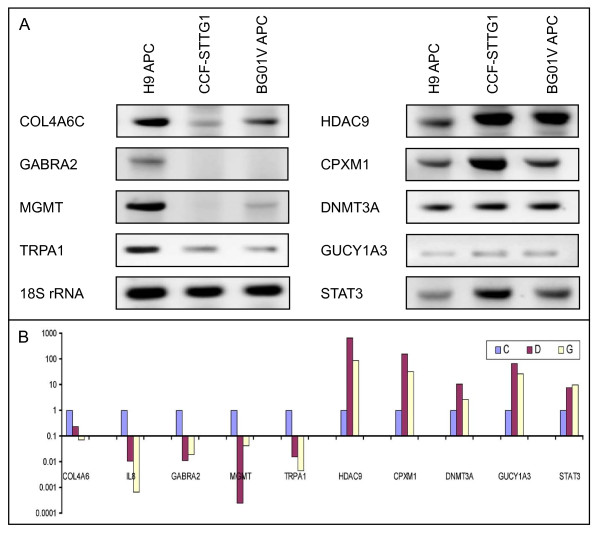
**Similarities in expression changes in trisomic APCs and astrocytoma cells were validated by RT-PCR analyses**. A. Semi-quantitative RT-PCR analysis shows relative under expression of *COL4A6*, *GABRA2*, *MGMT *and *TRPA1 *(left panels), and over expression of *HDAC9*, *CPXM1*, *DNMT3A, GUCY1A3 *and *STAT3 *(right panels) transcripts in trisomic BG01V APCs and CCF-STTG1 astrocytoma cells with respect to diploid H9 APCs. 18S RNA was used as a control. B. Quantitative RT-PCR validation of relative changes in expression levels of the transcripts shown in panel A in diploid H9 APCs (blue bars), trisomic BG01V APCs (yellow bars) and astrocytoma cells (red bars).

Both semi-quantitative and quantitative RT-PCR analyses were used to validate changes in expression levels of several transcripts detected by microarray analysis. Semi-quantitative RT-PCR analyses of nine transcripts that are over or under expressed in trisomic BG01V APCs and CCF-STTG1 astrocytoma cells relative to H9 APCs are shown in Figure 4A. Differential expression of transcripts identified by exon array analyses were also validated by qRT-PCR analyses using forward primers flanking unique exon junctions in conjunction with exon-specific reverse primers (Figure 4B). A success rate of greater than 93% was obtained for qRT-PCR validation of genes detected when microarray data was filtered at *p *value < 0.02. Additional differentially expressed transcripts that were validated by qRT-PCR analyses are listed in Additional file 1, Table S1. Human Cancer Pathway Finder PCR Arrays were used as a third method to evaluate relative changes in gene expression levels in diploid H9 and trisomic BG01V APCs. The Cancer Pathway Finder Array analysis identified numerous cancer-associated genes exhibiting substantial over expression in trisomic BG01V APCs relative to diploid H9 APCs, including *CDC25A*, *IGF1*, *MMP9*, *FGFR2*, *BRCA1*, *CASP8 *and *TERT1 *(Additional file 3, Figure S1).

### Trisomic APCs express markers of astrocytic cancer cells

**Figure 5:**
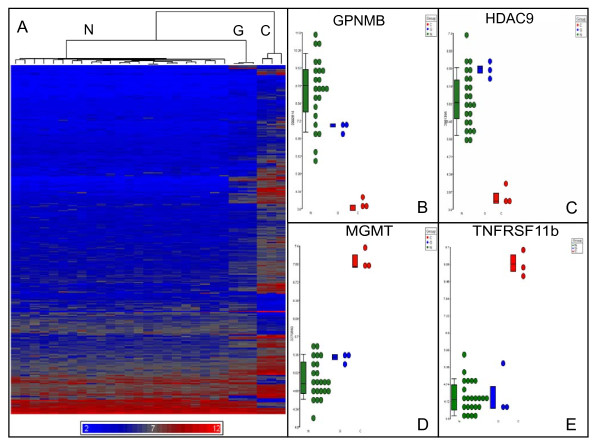
**Gene expression profile of trisomic BG01V APCs is similar to glioblastomas**. A. Heat map of unsupervised hierarchical cluster analysis of microarray hybridization data derived from the twenty-three glioblastoma patient samples (N), the three trisomic BG01V APC samples (G) and the three diploid H9 APC samples (C). Relative over expression is indicated by red and under expression by blue in the heat map. Data filtered using a *p *value of < 0.001. Individual gene dot plots displaying changes in mean expression levels of four transcripts: *GPNMB *(panel B), *HDAC9 *(panel C), *MGMT *(panel D) and *TNFRSF11b *(panel E). Each panel shows the individual hybridization data for all samples from each of the three groups, including the twenty-three glioblastoma samples (green), the three biological replicates of trisomic BG01V APCs (blue) and the three independent replicates of diploid H9 APCs (red).

Similarities in global gene expression profiles of BG01V APCs and CCF-STTG1 astrocytoma cells might arise from *in vitro *culture conditions used to direct astrocytic differentiation and/or from some characteristic of the CCF-STTG1 astrocytoma cell line used for individual pair wise and group comparisons. To exclude these possibilities, H9 and BG01V APCs expression profiles were also compared to twenty-three human glioblastoma patient samples analyzed using the same microarray platform [8]. Because we are interested in identifying expression changes in astrocytomas, all microarray data of histopathologically confirmed oligodendroglioma samples were excluded from this analysis. The heat map of hierarchical clustering of all transcripts exhibiting significant differences in relative expression levels in glioblastoma samples, trisomic BG01V APCs and diploid H9 APCs, filtered at a *p *value of < 0.001, is shown in Figure 5. This unsupervised hierarchical clustering demonstrates an obvious and striking similarity in overall gene expression profiles of the glioblastoma patient samples (samples N) and trisomic BG01V APCs (samples G) that is markedly distinct from the expression profile of diploid H9 APCs (samples C). This notable similarity indicates that, following differentiation along astrocytic pathways, trisomic BG01V APCs display a global gene expression profile that is more similar to human astrocytomas than to normal, diploid APCs. Examples of relative changes in individual expression levels of four genes are shown in Figure 5. Transcripts encoding the transmembrane glycoprotein, *GPNMB*, (Figure 5B) and histone deacetylase 9, *HDAC9 *(Figure 5C), are both over expressed in trisomic BG01V APCs and glioblastoma samples relative to diploid H9 APCs. In contrast, the O-6-methylguanine-DNA methyltransferase gene, *MGMT *(Figure 5D), and the tumor necrosis factor receptor family member, *TNFRSF11b *(Figure 5E), are both under expressed in aneuploid astrocytic-like cells relative to diploid H9 APCs.

**Figure 6:**
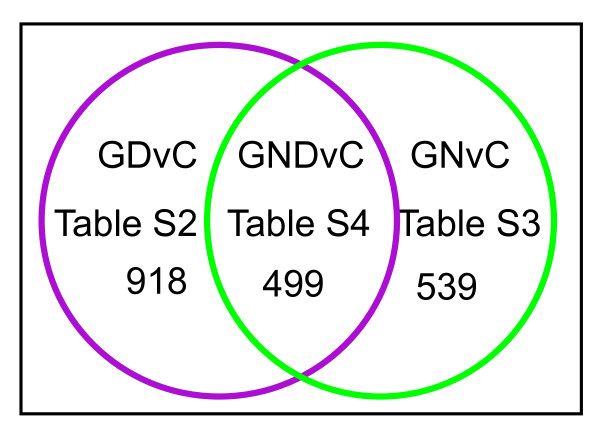
**Intersection of microarray data identifies markers of astrocytic cancer cells**. Venn diagram depicting the overlap between the two datasets, GDvC (Additional file 2, Table S2) and GNvC (Additional file 4, Table S3) that was used to identify the 499 transcripts exhibiting similar changes in expression patterns in all aneuploid astrocytic cell populations including trisomic BG01V APCs (samples G), glioblastoma patients (samples N) and CCF-STTG1 astrocytoma cells (samples D) relative to normal, diploid H9-derived APCs (samples C), which constitutes the GNDvC gene list shown in Additional file 5, Table S4.

To identify those transcripts exhibiting consistent sign changes in expression levels in both BG01V APCs (G) and glioblastoma patient samples (N) relative to H9 APCs (C), the intersection of the two data sets - GvC (2929 differentially expressed transcripts) and NvC (6611 differentially expressed transcripts) - was obtained by performing an ANOVA of the supervised hierarchical clustering (i.e. the group comparison). The intersect data set, GNvC, identifies 1,038 transcripts exhibiting statistically significant differential expression in both BG01V APCs and the twenty-three glioblastoma samples relative to H9 APCs (Additional file 4, Table S3). As illustrated in the Venn diagram in Figure 6, the intersection of Additional file 2, Table S2 (the GDvC group comparison) and Additional file 4, Table S3 (the GNvC group comparison), was used to further refine the gene list. The intersection of the two group comparisons identified 499 transcripts exhibiting consistent changes in expression levels in all classes of aberrant astrocytes, including trisomic BG01V APCs (G), glioblastoma samples (N) and CCF-STTG1 astrocytoma cells (D) relative to normal, diploid H9 APCs (C). The complete list of 499 transcripts is shown in Additional file 5, Table S4 (the GNDvC group comparison). A subset of the over expressed transcripts identified by this analysis, which exhibit at least three-fold over expression, is shown in Table 1 (GND > C). The finding of statistically significant and consistent over expression of these transcripts in all classes of aneuploid astrocytes including trisomic BG01V APC samples (G) glioblastoma patient samples (N) and cultured astrocytoma cell samples (D) suggests they encode markers characteristic of astrocytic cancer cells. This list includes, among others, *CSRP1*, *HSPA1A*, *TUBB2B*, *GPNMB*, *PSMD5*, *PTPRD*, *APOE*, *MAGEH1*, *TYW3*, *SESN3*, *CTSH*, *CRYZ*, *PIK3R1*, *TAF9B*, *GUCY1A3*, *GPRC5B*, *CPXM1*, *HDAC9*, *GUCY1B3*, *SIPA1L2*, *HNMT *and *THRB *many of which have been previously associated with cancer and some specifically with astrocytomas [10,11,13-20].

**Table 1:** Markers over expressed in astrocytic cancer cells (GND > C).

Gene symbol	Accession number	Fold change
*CSRP1*	NM_004078	57.34

*HSPA1A*	NM_005345	28.56

*TUBB2B*	NM_178012	19.70

*GPNMB*	NM_001005340	17.55

*PSMD5*	NM_005047	17.52

*PTPRD*	NM_002839	12.00

*APOE*	NM_000041	11.27

*MAGEH1*	NM_014061	10.26

*TYW3*	NM_138467	9.56

*SESN3*	NM_144665	8.03

*CTSH*	NM_004390	7.88

*CRYZ*	NM_001889	7.81

*PIK3R1*	NM_181523	6.50

*TAF9B*	NM_015975	6.17

*ETV1*	NM_004956	6.08

*GUCY1A3*	NM_000856	5.87

*GPRC5B*	NM_016235	5.84

*CPXM1*	NM_019609	5.38

*HDAC9*	NM_178423	5.15

*GUCY1B3*	NM_000857	4.76

*SIPA1L2*	NM_020808	4.47

*HNMT*	NM_006895	4.29

*SHMT1*	NM_004169	3.46

*THRB*	NM_001128177	3.13

A subset of gene transcripts derived from Additional file 5, Table S4 exhibiting at least three-fold over expression in trisomic BG01V APCs (G), glioblastoma samples (N) and CCF-STTG1 astrocytoma cells (D) relative to diploid H9 APCs (C), which was obtained by filtering data using a *p *value < 0.02. Fold change was derived from the GNvC group comparison in Additional file 5, Table S4.

### Trisomic APCs express markers of astrocytic cancer stem/progenitor cells

**Figure 7:**
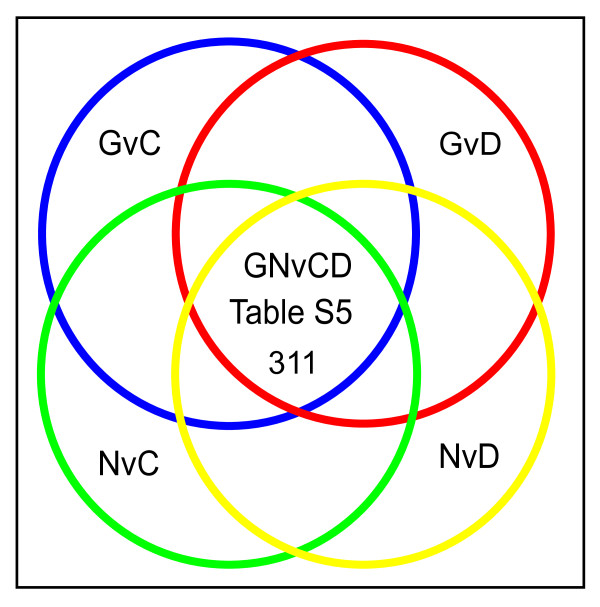
**Intersection of microarray data identifies markers of premalignant astrocytic stem-like/progenitor cells**. Venn diagram depicting the overlap between the four pair wise comparisons: GvC, GvD, NvC and NvD that was used to identify the 311 transcripts exhibiting similar changes in expression patterns in trisomic BG01V APCs (samples G) and glioblastoma patients (samples N) relative to diploid H9 APCs (samples C) and CCF-STTG1 astrocytoma cells (samples D), which constitutes the GNvCD gene list shown in Additional file 6, Table S5.

As indicated in Figure 6, 539 gene transcripts are differentially expressed in glioblastoma patient samples and BG01V APC samples relative to H9 APC samples (GNvC group) that are *not *also differentially expressed in the GDvC group comparison. Glioblastomas contain a heterogeneous mix of cells including rapidly proliferating malignant cells as well as slowly cycling cancer stem/progenitor cells. These putative brain tumor stem cells may comprise only a small percentage of the tumor mass. Markers of brain tumor stem cells are reportedly down regulated when glioblastoma cell lines are grown as adherent cultures [25,26]. We reasoned that markers of slowly-cycling, premalignant astrocytic stem/progenitor cells would be over expressed in the 23 glioblastoma samples (N) and trisomic BG01V APCs (G), but under expressed in the adherent CCF-STTG1 astrocytoma cell line (D) and H9-derived APCs (C). As illustrated in Figure 7, transcripts meeting these criteria are identified by performing an ANOVA of the intersection of the four individual pair wise comparisons - GvC, NvC, GvD and NvD - or the GNvCD group comparison. This group analysis identified 311 gene transcripts exhibiting significant differential expression when filtered using a *p *value of < 0.02 (Additional file 6, Table S5), of which approximately 75 transcripts were over expressed in both trisomic BG01V APCs (G) and glioblastoma samples (N) relative to diploid H9 APCs (C) and CCF-STTG1 astrocytoma cells (D). A subset of these over expressed transcripts, which are predicted to encode biomarkers of premalignant astrocytic stem/progenitor cells, is shown in Table 2 (GN > CD).

**Table 2:** Markers over expressed in premalignant astrocytic stem-like/progenitor cells (GN > CD).

Gene symbol	Accession Number	Fold change
*RGS5*	NM_003617	30.43

*CHI3L1*	NM_001276	25.26

*FKBP5*	NM_004117	10.71

*LPHN2*	NM_012302	10.33

*SLC40A1*	NM_014585	9.16

*IGFBP2*	NM_000597	8.42

*SERPINB9*	NM_004155	8.10

*PPP2R2B*	NM_181674	7.59

*PROM1*	NM_006017	7.34

*ST8SIA4*	NM_005668	7.23

*GPC4*	NM_001448	7.11

*P2RY5*	NM_005767	6.18

*LASS6*	NM_203463	5.41

*SNTB1*	NM_021021	5.24

*KCNMB4*	NM_014505	5.18

*ASTN1*	NM_004319	4.77

*CCDC3*	NM_031455	4.76

*RHOU*	NM_021205	4.74

*MTUS1*	NM_001001924	4.32

*PPAP2B*	NM_003713	3.60

*ZNF238*	NM_205768	3.53

A subset of gene transcripts over expressed in trisomic BG01V APCs (G) and glioblastoma samples (N) relative to diploid H9 APCs (C) and CCF-STTG1 astrocytoma cells (D). Fold change was derived from GNvCD group comparison in Additional file 6, Table S5, filtered at *p *value < 0.02.

**Figure 8:**
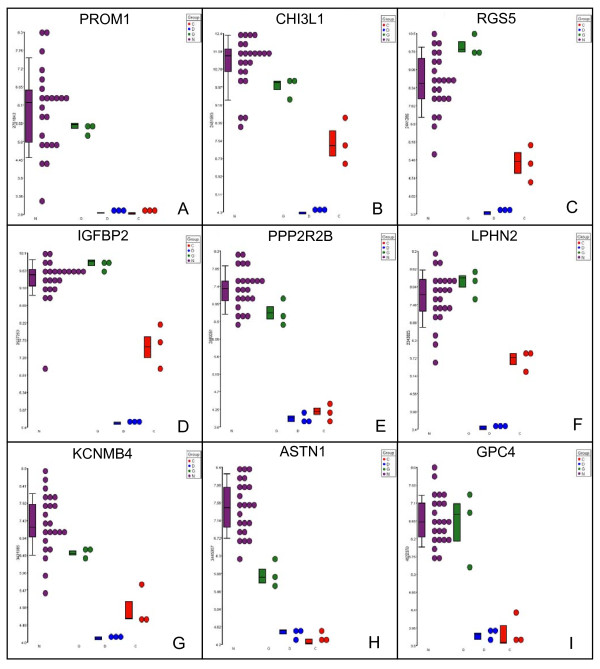
**Over expressed transcripts include known and novel markers of premalignant astrocytic stem/progenitor cells**. Individual gene dot plots showing expression levels of several over expressed transcripts in all twenty-three glioblastoma patient samples (purple, N), three trisomic BG01V APCs samples (green, G), three CCF-STTG1 astrocytoma cell samples (blue, D) and diploid H9 APCs (red, C). Transcripts shown are *PROM1 *(panel A), *CHI3L1 *(panel B), *RGS5 *(panel C), *IGFBP2 *(panel D), *PPP2R2B *(panel E), *LPHN2 *(panel F), *KCNMB4 *(panel G), *ASTN1 *(panel H) and *GPC4 *(panel I).

Individual gene dot plots displaying relative expression levels of several of the GN > CD transcripts are shown in Figure 8. Included is at least one transcript, *PROM1 *(Figure 8A), encoding the CD133 cell surface marker, which has been classified as a biomarker of brain tumor stem cells defined as those cells responsible for giving rise to rapidly proliferating, serial transplantable glioblastomas in immunocompromised mice [25,26]. *PROM1*, however, is neither the most statistically significant (*p *value of 4.08 × 10^-5^) nor the most abundantly over expressed (7.34 fold) transcript in Additional file 6, Table S5. Additional biomarkers indentified by the analysis exhibiting greater increases in relative expression levels (> 30 fold) and/or lower *p *values (< 7.90 × 10^-11^), include *CHI3L1 *(Figure 8B), *RGS5 *(Figure 8C) and *IGFBP2 *(Figure 8D), which have been shown to be over expressed in malignant astrocytomas - particularly recurrent glioblastomas that often contain a higher percentage of brain tumor stem cells [27,28] - suggesting that these may be more reliable biomarkers of brain tumor stem cells than CD133 (*PROM1*). The finding of significant over expression of biomarkers of brain tumor stem cells in the GN > CD data set suggests that this system can be used to refine the definition of a human astrocytic cancer stem/progenitor cell by identifying additional biomarkers that have not previously been associated with brain tumor stem cells. Individual dot plots of other over expressed transcripts identified in the GD > CD data set, including *PPP2R2B *(Figure 8E), *LPHN2 *(Figure 8F), *KCNMB4 *(Figure 8G), *ASTN1 *(Figure 8H) and *GPC4 *(Figure 8I) are shown.

## Discussion

In this study, an *in vitro *culture system was developed to differentiate diploid and trisomic hESCs into astrocytic progenitor cells (APCs), which were used to determine if gene expression profiles of trisomic APCs remain similar to, or deviate from, diploid APCs following astrocytic differentiation. The data indicate that expression profiles of trisomic BG01V APCs diverge considerably from diploid H9 APCs. Analysis of high-density microarray data revealed numerous, highly significant differences in transcript expression levels in trisomic BG01V APCs relative to diploid H9 APCs. Similar differences were observed when the human astrocytoma cell line, CCF-STTG1, was compared to diploid H9 APCs. Several expression level changes, initially detected by microarray analysis, were subsequently confirmed by qRT-PCR validation. A remarkably similar trend was observed when trisomic BG01V APCs were compared to human glioblastoma patient samples. Taken together, the data suggest that following differentiation along an astrocytic pathway trisomic BG01V APCs exhibit a global gene expression profile that is more similar to astrocytic cancer cells that to normal diploid hESC-derived APCs.

Although trisomic BG01V APCs continue to express markers of differentiated astrocytes, they are clearly distinct from diploid H9 APCs. Despite the high *PROM1 *expression in BG01V APCs when cultured under adherent conditions, there is insufficient evidence to classify trisomic BG01V APCs as brain tumor-initiating cells [25,26]. Although trisomic BG01V APCs were derived from multipotent neurospheres that give rise to oligodendrocytes or neurons under different culture conditions (Gopalakrishna-Pillai and Iverson, unpublished), it is also incorrect to consider BG01V APCs equivalent to transformed neural stem cells. Unlike the CCF-STTG1 astrocytoma cells, BG01V APCs do not readily dedifferentiate into non-adherent neurospheres (not shown). Thus, it may be more appropriate to consider trisomic BG01V APCs as one type of premalignant astrocytic stem/progenitor cell. BG01V APCs exhibit an increased rate of proliferation relative to diploid H9 APCs and display gene expression patterns more similar to the astrocytoma cell line and glioblastoma patient samples. Trisomic BG01V APCs may be predisposed to becoming astrocytic cancer cells, but have not yet acquired the full spectrum of mutations required for malignant transformation.

Because the BG01V trisomic hESC line studied here was not derived from the diploid H9 hESC line used for comparison, the results of this analysis cannot be used to identify those changes in gene expression that are predicted to be initiators of premalignant transformation or tumorigenesis (i.e. changes in gene expression that cause transformation). However, group analyses of the data sets can be used to identify differentially expressed transcripts in normal differentiated astrocytes/astrocytic progenitors relative to astrocytic cancer cells. For example, highly significant decreases in expression levels of several transcripts encoding known markers of normal astrocytes including *TRPA1*, *BDNF *and *MGMT *were detected by the analyses. Down regulation of *MGMT *transcript levels, via hypermethylation of the *MGMT *promoter, is associated with malignant progression of astrocytomas and is being used in the clinic to identify patients that may benefit the most from treatment with temozolomide [29,30]. Thus, transcripts exhibiting highly significant over expression in all classes of abnormal astrocytes, including trisomic BG01V APCs, CCF-STTG1 astrocytoma cells and glioblastoma patient samples with respect to diploid H9 APCs (Table 1, GND > C) may be diagnostic markers of transformation and/or potential therapeutic targets (i.e. changes in gene expression that are consequences of transformation). This list includes cell surface expressed markers such as *GPRC5*, signaling molecules such *PIK3R1 *and the histone deacetylase, *HDAC9*, for which small molecule inhibitors are currently under investigation in glioblastoma clinical trials [13,31].

Neural stem cells with astrocytic character arising in the sub ventricular zone are thought to be one source of origin of gliomas [32,33]. Donor-derived neural stem cells were recently demonstrated to give rise to tumors of glioneuronal origin in an Ataxia Telangiectasia patient [34]. Slowly cycling cancer stem cells, which are refractory to conventional radiation and chemotherapy, are thought to be a source of both the original tumor as well as recurrent tumors. These astrocytic cancer stem cells create an obstacle for effective treatment of malignant gliomas, yet identification of molecular markers characteristic of brain tumor stem cells is a major challenge complicated by their low numbers, elusive nature, heterogeneity of brain tumors and the difficulty of obtaining abundant quantities of normal human astrocytes that can serve as controls for global expression analyses [32]. As a result, gene expression profiles of rare subpopulations of brain tumor stem cells cannot be readily identified by microarray analyses of brain tumor samples. The system used in this study describes methods for both obtaining sufficient quantities of suitable control cells via directed differentiation of hESCs and using gene expression profiles of these cells to refine the list of putative biomarkers of the astrocytic cancer stem cells through rigorous bioinformatic analyses of the microarray data.

Given their astrocytic, premalignant and stem-like properties, the class of transcripts predicted to be biomarkers of premalignant astrocytic stem/progenitor cells are the GN > CD transcripts (Table 2, Additional file 6, Table S5, Figure 8), and include transcripts encoding numerous potential therapeutic targets and/or cell-surface expressed proteins. In addition to *PROM1*, *CHI3L1 *was also identified by the analysis. CHI13L1 (YKL-40) is expressed in a small percentage of glioblastoma cells upon initial diagnosis, but exhibits profound up regulation upon tumor recurrence [27]. The regulator of G protein signaling 5, RGS5, is over expressed in highly angiogenic astrocytomas and RGS5 expression is specifically up regulated in the vasculature of premalignant lesions [35]. FKBP5 is over expressed in gliomas and down regulation of FKBP5 expression using siRNAs suppresses glioma cell growth [36]. IGFBP2 over expression has been demonstrated to promote glioma growth as well as progression from low to high grade in mouse models [37]. Over expression of transcripts encoding transmembrane proteins KCNMB4, a neural-specific β subunit of a large-conductance, calcium-sensitive potassium channel associated with glioma cell growth [38], and LPHN2, a putative G-protein coupled receptor, were both identified by the comparative microarray analysis. As were ASTN1, an adhesion molecule associated with neuronal migration along astroglial fibers [39], and GPC4, a cell-surface expressed proteoglycan that may play a role in controlling cell division [40]. Additional over expressed transcripts identified by comparative global microarray analysis using *in vitro *differentiated trisomic BG01V APCs include those encoding signaling molecules such as the protein phosphatase, PPP2R2B, the purinergic receptor, P2RY5, the ras homolog, RHOU, and others not previously associated with astrocytomas or astrocytic cancer stem cells.

Inherent genetic instability of cultured hESCs renders them susceptible to gain or loss of entire chromosomes and/or discrete chromosomal regions. Gain of chromosomes 12 or 17 has been reported in several other hESC lines [2,3,7]. Amplification or deletion of discrete regions of chromosome 20 and 5 as well as mosaic gain of chromosome 12 has also been reported to arise spontaneously in cultured hESC lines [6]. Significantly, neural stem cells derived from some of these hESC variants have been clearly demonstrated to exhibit several features of neoplastic transformation *in vivo*. Thus, it is highly unlikely that trisomic BG01V hESCs line are unique in their ability to differentiate into premalignant astrocytic stem/progenitor cells upon *in vitro *directed differentiation. The *in vivo *evidence of neoplastic transformation of differentiated hESC variants suggests that the propensity toward transformation may be a somewhat common occurrence and underscores the absolute necessity of subjecting all hESC-derived cells to functional characterization prior to their use in therapeutic regimens [6]. Although BG01V hESCs may not be unique in exhibiting features of premalignant transformation following differentiation, the conspicuous differences in expression profiles of BG01V APCs and H9 APCs, combined with the striking similarities in expression profiles of BG01V APCs and glioblastoma samples suggest that gain of chromosomes X, 12 and/or 17 may be one of several routes by which transformation can be initiated in astrocytic progenitor cells. However, we cannot rule out the possibility that genetic events other than trisomy played a role in the initiation of premalignant transformation observed here since the trisomic hESC line, BG01V, is not a derivative of the diploid hESC line, H9.

That a constellation of gain-of-function and/or loss-of-function mutations in multiple genes is required for malignant transformation has been known for decades [41,42]. Aneuploidy, however, has been associated with cancer for more than a century [43]. Given the high-degree of aneuploidy observed in glioblastoma patient samples, it is difficult to distinguish those genes or chromosomal regions associated with tumor initiation or propagation from those representing random events arising from the inevitable genetic instability common to these high-grade tumors. Comprehensive analysis of chromosomal aberrations in 141 glioma samples identified approximately 35 broad and focal regions of gene amplifications and deletions demonstrating statistically significant associations in human gliomas [44], and revealed that amplification of a number of chromosomal regions, including chromosomes 12 and 17, met the threshold for significance in these glioma samples, including secondary glioblastomas arising from low-grade gliomas. High-resolution copy number analysis of glioma samples also revealed recurrent gain of multiple sub-regions of chromosome 12 in secondary glioblastomas arising from lower grade astrocytomas [45]. Since recurrent gain of chromosomes 12 or 17 has been observed in a number of karyotypically abnormal hESC lines [2-4,7], this also suggests that other aneuploid hESC variants might exhibit properties similar to trisomic BG01V cells upon differentiation into astrocytes.

Dot plots for several additional markers associated with gliomas are shown in Additional file 7, Figure S2. Amplification of *EGFR *(or gain of chromosome 7) is often seen in high-grade gliomas [11]. Not surprisingly, over expression of *EGFR *in some glioblastoma samples is also observed here (Additional file 7, Figure S2, panel A). Loss of chromosome 10 (or regions of chromosome 10 encoding the *PTEN *locus) is associated with some *de novo *glioblastomas, and reduced *PTEN *expression in some glioblastoma samples is seen here (panel B). *IL13RA2 *is reportedly over expressed in ~90% of glioblastoma samples. Exon microarray analysis indicates bifurcation of patient samples into two distinct groups; one group exhibits significant over expression of *IL13RA2 *transcripts, whereas *IL13RA2 *is expressed at levels similar to controls in the other group of patient samples (D). Relative expression levels of *PDGFB *(F), *PDGFRA *(G) *PDGFRB *(H) are also shown in Additional file 7, Figure S2. Although median expression level of *PDGFRB *is decreased relative to controls, there is a wide range of expression levels, which may also reflect distinct glioma subtypes. Mutations in a number of genes have also been associated with gliomagenesis, although many are somatic mutations that do not necessarily result in expression level changes [11]. Several are shown in Additional file 7, Figure S2, including *AKT1 *(C), *NRG1 *(E), *TP53 *(I), *MDM2 *(J), *NF1 *(K) and *RB1 *(L). There is no obvious correlation in relative expression levels of these markers in glioblastoma samples and/or premalignant astrocytic progenitors, with perhaps the exception of *NRG1 *(*HER2 *ligand) where expression levels are lower in all samples relative to the diploid H9 APCs. Although trisomy for chromosomes 12 and 17 may be one potential precipitating event resulting in transformation of BG01V hESCs into premalignant APCs during differentiation, not all differences in relative gene expression levels can be explained by the trisomy. Dot plots for a number of genes that are known to be expressed in astrocytes, known to be associated with cancer and known to map to chromosomes 12 or 17 are also shown in Additional file 7, Figure S2, including *STAT3 *(panel M, chromosome 17) where expression levels in glioblastoma, BG01V APCs and CCF-STTG1 samples are higher than H9 APCs, *ERBB2 *(*HER2 *- panel N - chromosome 17) where expression levels are higher in BG01V APCs and glioblastoma samples but also high in diploid H9 APCs, and both *RARA *(panel O, chromosome 17) and *RARG *(panel P, chromosome 12), where expression levels are higher in BG01V APCs relative to H9 APCs but also lower in glioblastoma samples. Thus, not all transcripts encoded by genes that map to chromosomes X, 12 and/or 17 are over expressed in BG01V APCs and not all over expressed gene transcripts in BG01V APCs map to chromosomes X, 12 and/or 17. We think that most gene transcripts that are over expressed in BG01V APCs relative to H9 APCs are consequences of premalignant transformation rather than causes of premalignant transformation.

Nonetheless, gain of chromosomes X, 12 and/or 17 has also been observed in carcinoma in situ of the testis, which is thought to be the common pre-invasive progenitor cell (or precancerous stem cell) that gives rise to both seminoma and non-seminomatous testicular germ cell tumors [46]. Recurrent amplification of 17q23.2 in some breast tumors has recently been reduced to a 249 kb minimal region including potential tumor driver genes, *RPS6KB1 *and mir-21 [47]. Finding gains of similar chromosomal regions in tumors whose origins are unrelated to the nervous system suggests that trisomy for these chromosomal regions is a type of moderate aneuploidy common to other precancerous progenitor or stem cells and hypothesized to be an initiator of tumorigenesis [48-50]. This suggests that directed differentiation of trisomic hESC variants along other lineages might be used to simulate early molecular events occurring enroute to malignant transformation of other cancers and to identify diagnostic markers and/or molecular targets amenable to therapeutic intervention.

## Conclusions

An *in vitro *culture system was developed in which diploid and trisomic hESCs were differentiated into homogenous populations of human astrocytic progenitor cells (APCs) suitable for global gene expression profiling using high-density microarrays. Expression profiles of the hESC-derived APCs were compared to a malignant astrocytoma cell line and glioblastoma tumor samples, and used to demonstrate that trisomic APCs share many properties with the astrocytoma cell line and glioblastoma samples. The bioinformatic analysis employed here facilitated identification of numerous differentially expressed transcripts that are characteristic of astrocytic cancer cells. This analysis was also used to identify biomarkers of the subpopulation of astrocytic cancer stem cells that comprise only a small fraction of diverse cell types found in heterogeneous brain tumors. Directed differentiation of trisomic hESCs is a powerful *in vitro *model system for investigating changes in gene expression occurring enroute to malignant transformation and for identifying molecular markers characteristic of premalignant stem/progenitor cells that may be amenable for therapeutic intervention for patients with astrocytomas. The results of this analysis further underscore the need for exercising extreme caution when utilizing stem cells in regenerative medicine.

## Abbreviations

Non standard abbreviations used in the manuscript include hESC: (human embryonic stem cell); APC: (astrocytic progenitor cell).

## Competing interests

Invention disclosures describing the *in vitro *culture system and bioinformatic analysis used here to identify putative biomarkers of premalignant/malignant stem-like/progenitor cells in aneuploid hESCs and APCs have been filed; there is currently no patent pending.

## Authors' contributions

SG-P contributed to the conception and design of the experiments, data acquisition, data analysis and interpretation and final approval of manuscript. LEI contributed to the conception and design of the experiments, data analysis and interpretation, manuscript writing and final approval of manuscript.

## Pre-publication history

The pre-publication history for this paper can be accessed here:

http://www.biomedcentral.com/1755-8794/3/12/prepub

## Supplementary Material

Additional file 1 **Table S1: RT-PCR Primer Sequences**. Sequences of forward and reverse primers used for qRT-PCR validation and list of all transcripts where changes in expression levels, first detected by microarray analysis, were subsequently validated by qRT-PCR analysis. Click here for file

Additional file 2 **Table S2: Differentially expressed gene transcripts in GD v. C cell populations**. Excel spreadsheet of all differentially expressed genes in trisomic BG01V APCs (samples G) and CCF-STTG1 astrocytoma cells (samples D) relative to H9 APCs (samples C) and referred to in the text as the GDvC comparison. All data filtered using a *p *value of < 0.02. Click here for file

Additional file 3 **Figure S1: RT-PCR analysis using Human Cancer Pathway Finder PCR Arrays**. The four panels on the left show quantitative changes in expression levels of all cancer-associated gene transcripts included in the Cancer Super Arrays. The heat map displaying relative over expression (red) or under expression (green) of these cancer-associated gene transcripts in samples C (diploid H9 APCs) and samples G (trisomic BG01V APCs) is shown in the right panel. Click here for file

Additional file 4 **Table S3: Differentially expressed gene transcripts in GN v. C cell populations**. Excel spreadsheet of all differentially expressed genes in BG01V APCs (samples G) and glioblastoma patient samples (samples N) relative to H9 APCs (samples C) and referred to in the text as the GNvC comparison. All data filtered using a *p *value of < 0.02. Click here for file

Additional file 5 **Table S4: Differentially expressed gene transcripts in astrocytic cancer cells (GND v. C)**. Excel spreadsheet of all genes exhibiting differential expression patterns in BG01V APCs (samples G), glioblastoma patient samples (samples N) and CCF-STTG1 astrocytoma cells (samples D) relative to H9 APCs (samples C). The list contains the 499 genes identified (indicated in Figure 6) at the intersection of GDvC (Additional file 2, Table S2) and GNvC (Additional file 4, Table S3) and referred to in the text as the GNDvC comparison. All data filtered using a *p *value of < 0.02. Click here for file

Additional file 6 **Table S5: Differentially expressed gene transcripts in premalignant astrocytic stem-like/progenitor cells (GN v. CD)**. Excel spreadsheet of the 311 differentially expressed genes (indicated in Figure 7) in BG01V APCs (samples G) and glioblastoma patient samples (samples N) relative to CCF-STTG1 astrocytoma cells (samples D) and H9 APCs (samples C) and referred to in the text as the GNvCD comparison. All data filtered using a *p *value of < 0.02. Click here for file

Additional file 7 **Figure S2: Transcript expression profile of genes associated with glioblastomas and/or genes that map to trisomic chromosomes**. Dot plots of relative expression levels of several gene transcripts located on trisomic chromosomes (12 or 17) and several genes associated with brain tumors are shown for glioblastoma patient samples (purple, N), trisomic BG01V APCs (green, G), CCF-STTG1 astrocytoma cells (blue, D) and diploid H9 APCs (red, C). Transcripts shown are *EGFR *(panel A), *PTEN *(panel B), *AKT1 *(panel C), *IL13RA2 *(panel D), *NRG1 *(panel E), *PDGFB *(panel F), *PDGFRA *(panel G), *PDGFRB *(panel H), *TP53 *(panel I), *MDM2 *(panel J), *NF1 *(panel K), *RB1 *(panel L), *STAT3 *(panel M), *ERBB2 *(panel N), *RARA *(panel O) and *RARG *(panel P). Click here for file
