# Supplementary material for: Astrocytes derived from trisomic human embryonic stem cells express markers of astrocytic cancer cells and premalignant stem-like progenitors
Source: BMC Med Genomics. 2010 Apr 27;3:12. doi: 10.1186/1755-8794-3-12 (PMC2873256; doi:10.1186/1755-8794-3-12)
Supplement: Additional file 1 — Table S1: RT-PCR Primer Sequences. Sequences of forward and reverse primers used for qRT-PCR validation and list of all transcripts where changes in expression levels, first detected by microarray analysis, were subsequently validated by qRT-PCR analysis. [file 1755-8794-3-12-S1.DOC]

**Table S1. RT-PCR validated gene transcripts and primer sequences.**

| Semi-quantitative RT-PCR | | |
| --- | --- | --- |
| Gene | Forward primer | Reverse primer |
| *LIN28* | ATCTGTAAGTGGTTCAACGTGCGCA | CACTAGGGCCCTGCTGGGCCTTCAG |
| *FGFR1* | CTTCTGGGCTGTGCTGGTCACAGCCAC | ACTGGAAGGGCATTTGAACTTCACTG |
| *GFAP* | AGTCCCTGGAGAGGCAGATGCGCGAGC | ATGTTCCTCTTGAGGTGGCCTTCTGAC |
| *MGMT* | CGCTTTGCGTCCCGACGCCCGCAGGTCC | TGCCTGCCAGGGCTGCTAATTGCTGG |
| *TRPA1* | TATCTTCAATGTCCATTAGAATTCACC | ATGAGACCAAGACAGTAAGATCC |
| *DNMT3A* | GCGTGGATCGTAGCCTGAAAGACG | GAGGCATTCTTGTCCCCACGATCG |
| *HDAC9* | AACGAAAGACACTCCAACTAATGG | GGAAGATGCCGGGATGCTGCCTCC |
| *18SRNA* | CGGGCGCCGGCGGCTTTGGTGAC | GGTCACCATGGTAGGCACGGCGAC |
| *GABRA2* | AGGTTGCTCCTGATGGCTCTAGG | TCTTGGAGATGGTGGAGAGAACTGG |
| *CPXM1* | ACCACCAAGGTCCCAGGCTCG | CTCCGGCAGGAGGTTCAGCACTGG |
| *COL4A6* | CCCACAAGGAGCACCCGGATTTCC | CCATGAATTCCAGCTCTCCAGTAG |
| *GUCY1A3* | TACTCATGCTGTTCAGATAGCGCTG | AGAGTGCAGTCCAATTCGCATCTTG |
| *STAT3* | CCAGGCACCTTCCTGCTAAGATTCAG | TAGCGCACTCCGAGGTCAACTCCATG |
| *NOGGIN* | GTGAAGACCTGGTTCCAGAACCAG | TCCAAGTCACTGGCAGGAGAATTTGG |
|  | | |
| Real-time (quantitative) RT-PCR | | |
| Gene | Forward primer | Reverse primer |
| *COL4A6* | CCCACAAGGAGCACCCGGATTTCC | GACCAGGAGGCCCTGGAGGACCTTG |
| *IL8* | ATGTCAGTGCATAAAGACATACTCC | TCAGAAAGCTTTACAATAATTTCTG |
| *MGMT* | GTGCGCACCGTTTGCGACTTGGTAC | TTTATTTCGTGCAGACCCTGCTCAC |
| *TRPA1* | GATGAATGTCTTAAGATTTTCAGTC | CATGCAGAAATCTAAAAGTACCTTC |
| *HDAC9* | CCACCACACATCATTGGATCAAAGC | CCTTCAAGTTGGGCTCAGAGGCAG |
| *CPXM1* | TCGCGCAGCCCGGGACCACCAAGG | ACATGCTGTTCTGAGGTCCCGTTAG |
| *DNMT3A* | CCCTTCTTCTGGCTCTTTGAGAATG | CATCAATCATCACAGGGTTGGACTC |
| *GUCY1A3* | TACTCATGCTGTTCAGATAGCGCTG | AGAGTGCAGTCCAATTCGCATCTTG |
| *STAT3* | TCCACTGGTCTATCTCTATCCTGAC | ATGGTATTGCTGCAGGTCGTTGGTG |
| *GABRA2* | CGTCAAGATCAGGGCAAAAGGAAG | TTTGTCTTCATCACCGCCGCTCTG |
| *NEGR1* | TCGGTGTGAAACCAGTTCTGAATAC | TTAATGCGAAGTTCTGACTTCCCTG |
| *TSPAN15* | TTGGAGCAAGAATCAGTACCACGAC | CACATGGTGTTGACAACTTCTGTCG |
| *ANXA10* | CTGATTGGGGATATGAGGGAGCAGC | TTCTCATCAGTGCCTACTCCCTTC |
| *RHOJ* | AACCCTGCCTCTTACCACAATGTCC | TTGGGTCATCACGGAGATCAATCTG |
| *TNFRS11B* | GTGATGAGCGCACGGGCTGCGGAG | CTTAATGGAGATGTCCAGAAACACG |
| *CXCL5* | TCCTTCGAGCTCCTTGTGCGCGCTG | CACAGCAGCGGCAGGACCAGCGCTG |
| *BST1* | TGGGAAGCCTTTAAAGTGGCGCTGG | GTGGCTATTTTCCCAGAACAGGGAC |
| *CXCL6* | ACTATGAGCCTCCCGTCCAGCCGCG | AGCACAGCAGAGACAGGACCAGCGC |
| *TNFRS10D* | CACGACCAGAGACACCGTGTGTCAG | CTTGACCATCCCTCTGGGACACCCTG |
| *RIPK2* | CATATGATATACCTCACCGAGCACG | GTTCAAGTTCTATTAAACATTTTAAG |
| *CXCL1* | GAGCGTCCGTGGCCACTGAACTG | GCAAGCTTTCCGCCCATTCTTGAGTG |
| *COLEC12* | ACGACTTCGCAGAGGAGGAGGAGGTG | CTTTATATCCCAAAATGGCTACTGTG |
| *CDH6* | CCGTGGAGCCCGAGTCTGAATTCATC | TCATCTGCATCCGTCGCAGTGACTTGG |
| *PAPPA* | CCACCCTGCCTTCGTGAAGAAGCAGC | GAATGTTCTTCAGCTCATTAACATCC |
| *DNMT3B* | TGTAATCCAGTGATGATTGATGCC | CATTCTTTGATGCTATCACGGGCCTG |
| *FAT* | AAACCTCTTGATGCAGAACAGAAGTC | TCTATTACTTTGATGAATACCTGAG |
| *RGS18* | ACTAAGTCTTCTTGTGCAGAAACCTG | ACTGCCTCTTCAGGGGAGACTCTTG |
