# Supplementary figures and images for: Astrocytes derived from trisomic human embryonic stem cells express markers of astrocytic cancer cells and premalignant stem-like progenitors
Source: BMC Med Genomics. 2010 Apr 27;3:12. doi: 10.1186/1755-8794-3-12 (PMC2873256; doi:10.1186/1755-8794-3-12)

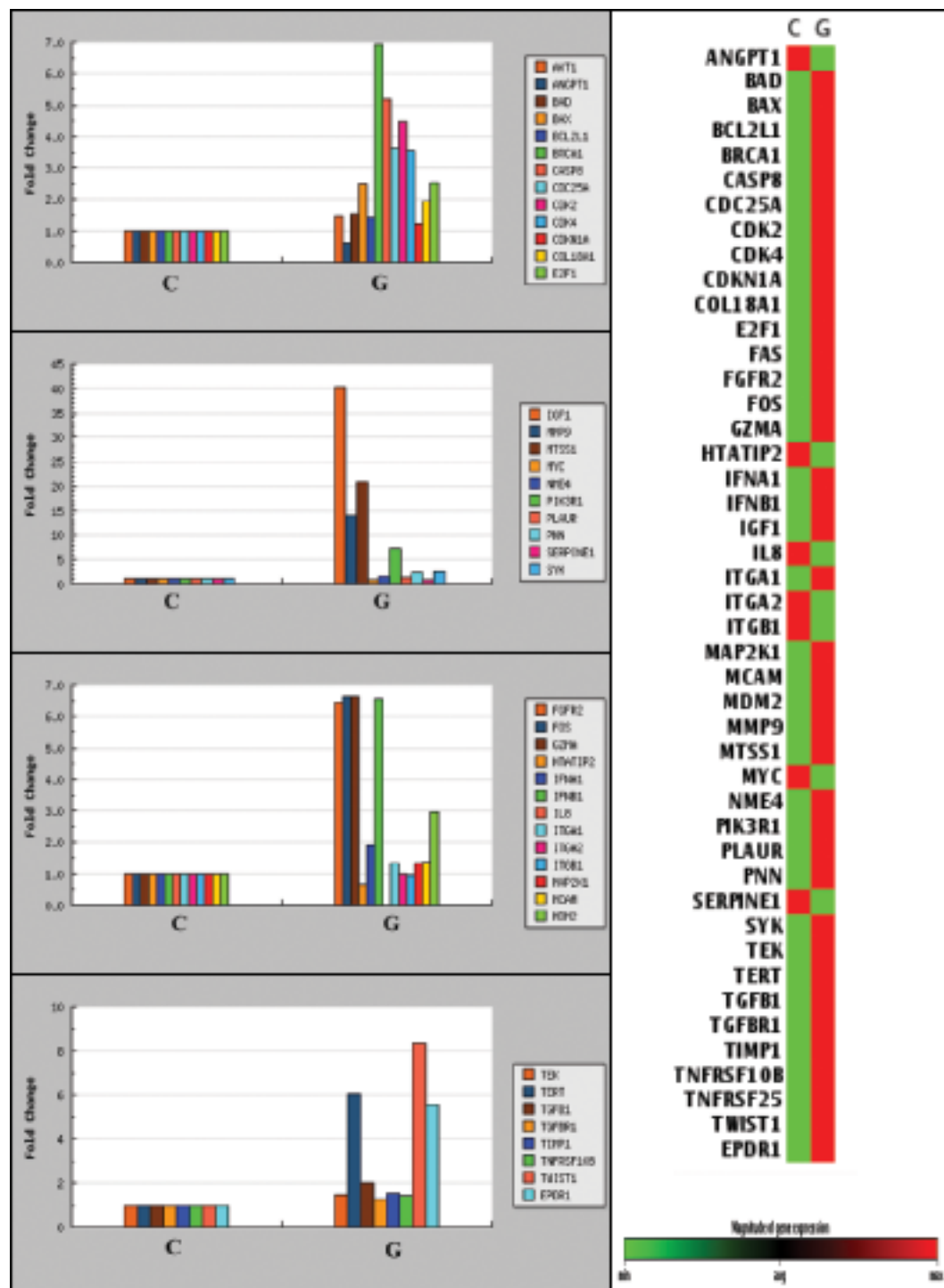

Supplement: Additional file 3 — Figure S1: RT-PCR analysis using Human Cancer Pathway Finder PCR Arrays. The four panels on the left show quantitative changes in expression levels of all cancer-associated gene transcripts included in the Cancer Super Arrays. The heat map displaying relative over expression (red) or under expression (green) of these cancer-associated gene transcripts in samples C (diploid H9 APCs) and samples G (trisomic BG01V APCs) is shown in the right panel. [file 1755-8794-3-12-S3.PDF]

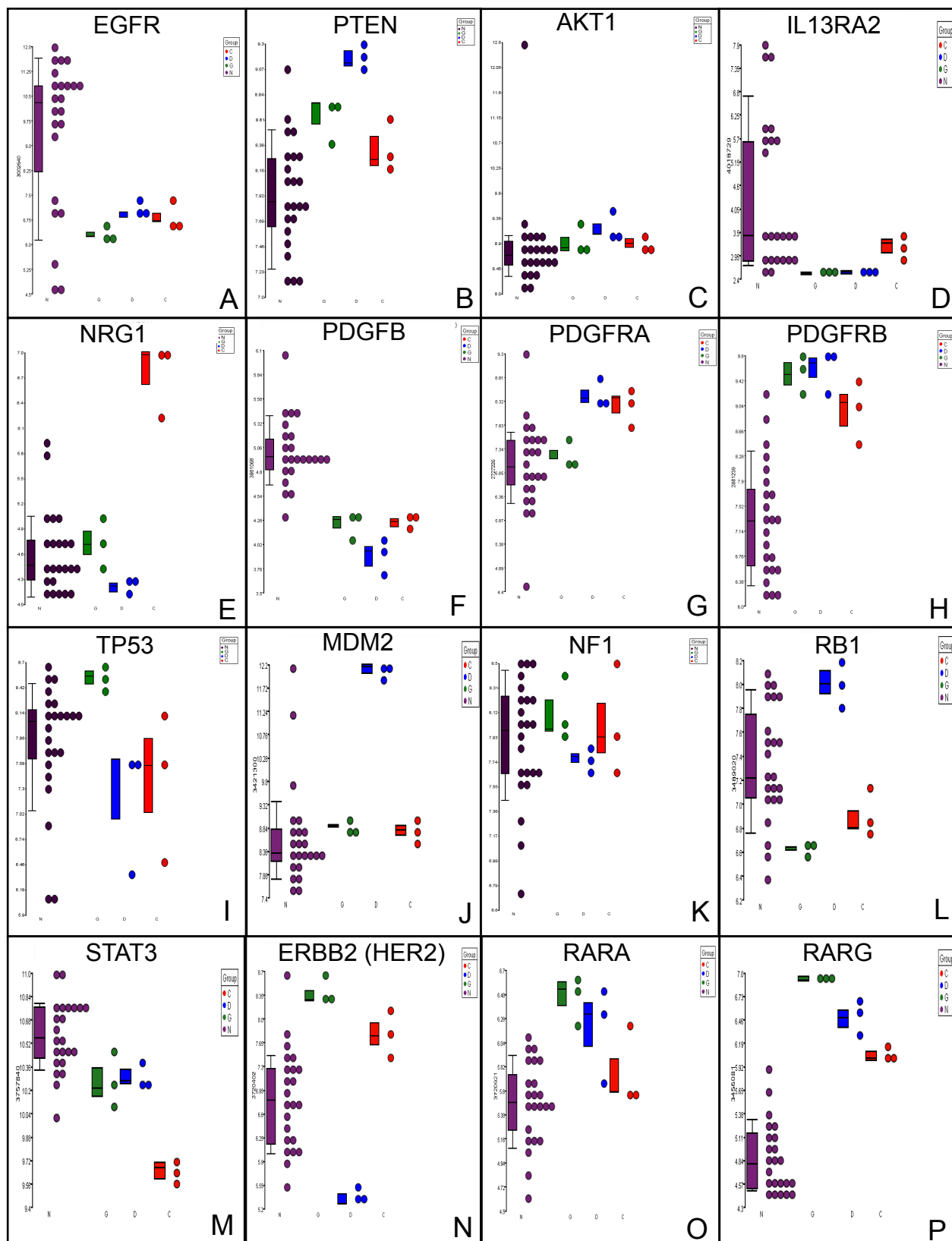

Supplement: Additional file 7 — Figure S2: Transcript expression profile of genes associated with glioblastomas and/or genes that map to trisomic chromosomes. Dot plots of relative expression levels of several gene transcripts located on trisomic chromosomes (12 or 17) and several genes associated with brain tumors are shown for glioblastoma patient samples (purple, N), trisomic BG01V APCs (green, G), CCF-STTG1 astrocytoma cells (blue, D) and diploid H9 APCs (red, C). Transcripts shown are EGFR (panel A), PTEN (panel B), AKT1 (panel C), IL13RA2 (panel D), NRG1 (panel E), PDGFB (panel F), PDGFRA (panel G), PDGFRB (panel H), TP53 (panel I), MDM2 (panel J), NF1 (panel K), RB1 (panel L), STAT3 (panel M), ERBB2 (panel N), RARA (panel O) and RARG (panel P). [file 1755-8794-3-12-S7.PDF]
